# The investigation of type and concentration of bio-aerosols in the air of surgical rooms: A case study in Shariati hospital, Karaj

**DOI:** 10.1016/j.mex.2019.03.016

**Published:** 2019-03-19

**Authors:** Zahra Tolabi, Mahmood Alimohammadi, Mohammad Sadegh Hassanvand, Ramin Nabizadeh, Hamed Soleimani, Ahmad Zarei

**Affiliations:** a Department of Environmental Health Engineering, School of Public Health, Tehran University of Medical Sciences, Tehran, Iran; b Department of Environmental Health Engineering, School of Public Health, Gonabad University of Medical Sciences, Gonabad, Iran

**Keywords:** Type and concentration of bio-aerosols, Bioaerosol, Bacteria, Fungi, Indoor air pollutant, Hospital infections, Aspergillu

## Abstract

The presence of bio-aerosols is one of the main causes of hospital infections that can be dangerous especially for immunocompromised patients. This research aimed to determine the relationship between hospital infections and surgical incision size in addition to determining the concentration and bacterial and fungal bioaerosols found in the operating rooms. This cross-sectional descriptive-analytic study was carried out in the operating rooms of Shariati Hospital in Karaj, Iran during the years 2016 and 2017. A total of 198 bacterial samples and 198 fungal samples were collected and analyzed using a passive sampling standard method (1/1/1) for 180 days. Tryptic Soy Agar (TSA) and Sabouraud Dextrose Agar (SDA) medium were used for bacterial and fungal samples, respectively. Relevant differential tests were used to determine the genus and species of bacteria and fungi such as DNase test, Bile-esculin, motility test urease test. In general, this work presents:

• The present evaluated the relationship between bioaerosols concentration and surgical incision size.
• The intraoperative concentration of bacterial and fungal bioaerosols in indoor air of the orthopedic, internal and cesarean operating rooms was significantly higher than their preoperative concentration (p-value<0.05).
• Also, there was not significant difference between the bacterial and fungal concentrations in various operating rooms) p-value<0.05).

## Graphical abstract

**Figure:**
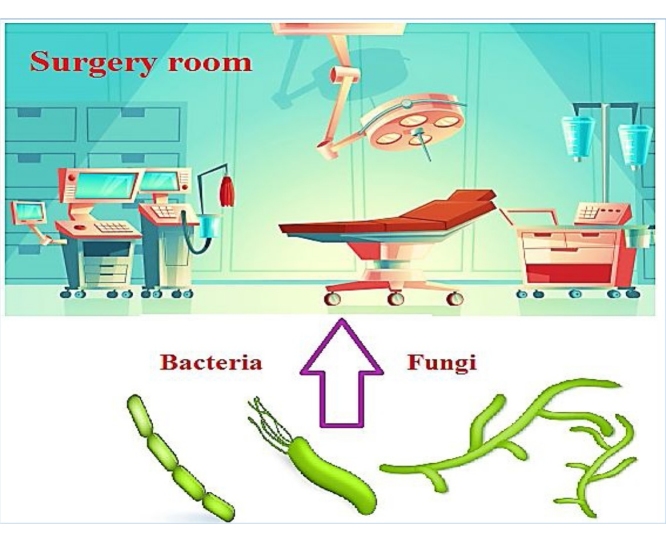

**Specifications Table**

**Table:** 

**Subject Area:**	*Environmental health; Airborne pollutants*
**More specific subject area:**	*Bioaerosol*
**Method name:**	Type and concentration of bio-aerosols
**Name and reference of original method:**	*F. Ghanizadeh, H. Godini A review of the chemical and biological pollutants in indoor air in hospitals and assessing their effects on the health of patients, staff and visitors, Rev. Environ. health, 33 (2018), pp. 231-245* [23].
**Resource availability:**	*Data are presented within the article*

## Method details

Aerosols are among important air pollutants producing due to natural processes and human activities [1,2]. Human exposure to bioaerosols in indoor air causes many problems in health care centers. High concentrations of different bioaerosols species are reported in hospitals. Exposure to bioaerosols is associated with adverse health effects, including transmission of infectious diseases, acute toxic effects, and respiratory syndromes such as asthma and allergies [3]. The presence of bioaerosols in the hospital wards is one of the main causes of hospital infections and can be dangerous especially for immunocompromised patients [4]. Overall, hospital infections impose important health and economic problems on societies, with an estimated population of around 1.4 million people being affected at any given moment in the world [5].

Bioaerosols are contaminant particles that contain microorganisms such as bacteria and fungi spores and their related products (endotoxin, mycotoxin, etc.), viruses, pollen grains, fungi, plant residues, insect fragments, and human and animal skin cells [[6], [7], [8], [9]]. The bio-aerosol particles range from 0.001–100 μm in diameter [10]. Bioaerosols smaller than ten μm in diameter create more severe health complications, especially in hospitals [11]. Previous studies have attributed about 5–34% of indoor air contamination to the presence of bioaerosols. Respiratory symptoms and decreased lung function are one of the most critical health complications of exposure to bioaerosols [12]. Bacteria and fungi make up a wide range of microorganisms that enter the hospital indoor air through ventilation systems, visitors and patients [13,14]. Passive sampling is arguably the most readily available, economical, and unobtrusive method of bioaerosol sampling and relies on particles settling using gravity, on a collection substrate housed in a settle plate [15]. This method provides a risk assessment that leads to estimating the population of microorganisms in the air capable of depositing on critical surfaces [16]. The results of such sampling are known as the index of microbial air contamination (IMA) [17]. Since the survival of microorganisms in the active sampling method is inversely related to the velocity at which entry into the sampler, active sampling increases the survival probability of bioaerosols.

In recent years, the percentage of the effectiveness of hospital disinfection processes has been decreased due to the incidence of antimicrobial resistance in bacterial pathogens. Consequently, microorganisms existing on the disinfected surfaces are again suspended in the air and they are much likely to be inhaled by different individuals, especially patients [18]. Also, these air-borne microorganisms may enter the patient’s body and cause severe infections through open wounds, especially during the surgical procedure [16]. On the other hand, awareness of the prevalence of microbial flora in the hospital settings is essential in achieving a better understanding of the possible types of infections and allergies that may be caused by bioaerosols in the hospital indoor air. Considering the importance of the previous, there is a necessity to study the current state of the air quality of the operating rooms of hospitals in the country in terms of precipitate bioaerosols. The present study aimed to determine the concentration and bacterial and fungal bioaerosols found in the operating rooms of Shariati Hospital in Karaj, Iran as well as the effect of the surgical incision size on the concentration of bioaerosols during 2016–2017 using passive sampling method.

## Methodology

### Study area and sampling procedure

This cross-sectional descriptive-analytic study was conducted to investigate the concentration and types of bacterial and fungal bioaerosols in the operating rooms of Shariati Hospital in Karaj city, Iran between May 2016 and February 2017. Sampling was carried out in the indoor air of the surgery rooms of Shariati hospital. A total of 4 operating rooms (orthopedic, eye, cesarean and internal surgeries) of this hospital were used as the sampling site. In the internal surgical room because of less area than other operating rooms, sampling was done in two points, and in other operating rooms, the sampling was conducted in 3 points (entrance, near the surgical bed, the end of the operating room). Sampling was carried out for 180 days in 6 stages. In each stage, sampling was taken before surgery, during the surgery, and also after surgery.

### Sampling methods

We used passive (settle plate, according to the index of microbial air contamination (IMA)) sampling to collect indoor bioaerosols. Passive sampling method involves the placement of culture plates that are also known as settling plates in the Sampling environment. The plate lid was remained open for an hour and exposed to the indoor air to sample the bioaerosols suspended in ambient air [19]. The standard 1/1/1passive sampling method was used in the present. In this method, the following instructions should be done: Plates containing the culture media should be located at the height of 1 m above the ground level on a stool while maintaining at least one-meter distance from any physical barriers such as wall, windows and entrance door or any obstacle the surrounding obstacles and wall and also, sampling should take for 1 h [16]. Bacterial and fungal sampling was carried out using a 9-cm-diameter plate at each sampling point. These instructions were implemented for all samples. Finally, six sampling steps were performed in each of the operating rooms from June 2016 until December 2016. A total of 198 bacterial and 198 fungal samples were collected for 180 days. At the same time, the parameter of the surgical incision size also recorded. After sampling, the samples were transferred to the Microbiology Laboratory of Shariati Hospital in Karaj city, Iran in order to perform related analyses.

Sabouraud dextrose agar (SDA) and tryptic soy agar (TSA) culture media were used for fungi and bacteria, respectively. Cycloheximide (500 mg L−1) was used to inhibit bacterial growth in the fungal culture media, while chloramphenicol (100 mg L−1) was used to suppress fungal growth in the bacterial media. After the sampling, the culture media were placed in zip kips and transferred to the laboratory in a cool box [20,21].

### Identification of bacterial and fungal bioaerosols

After sampling, the bacterial culture media was transferred to the laboratory under sterile conditions, and then incubated for 2–4 days at 30 °C and fungal culture media were grown at 25–27 °C (lab temperature) for 70–120 h and some colonies were finally counted. Samples harvested from the culture media were tested in the laboratory to detect the types of airborne bacteria.

Differential tests used to detect bacteria initially included Catalase Testing, in which all bacteria were catalase-positive. At this stage, the bacteria were grown on enriched blood agar (BA) media, as well as a MacCONKEY agar. Blood agar media was used to analyze gram-negative bacteria. After 24–48 h, colonies grew on BA media, but no colonies were observed on the MacCONKEY agar media, which was also expected. Colonies cultured on BA media were subjected to differential tests, which included bacitracin and novobiocin susceptibility tests to differentiate Staphylococcus species, Danse test to investigate the presence of Staphylococcus aureus, Bile-esculin test to analyze the presence of enterococcus, motility test to study the presence of Bacillus anthracis, and also urease test to investigate the presence of diphtheria. Other tests that had been used was included the use of arginine and ornithine amino acids, mannitol test, mannose, maltose, etc. [22].

Several methods are used to carry out differential testing on different types of fungi, including the identification of the colony apparent characteristics and its microscopic forms. The apparent characteristics taken into account to detect the fungal colony included: the growth rate, the state and shape of the colony, such as flatness, prominence and regularity or irregularity, colony appearance, colony color (white, yellow, green, blue, cream, purple), the presence of a pigment, and the color behind the colony caused by fungal pigment production, which is released in the medium and causes such color [22,23].

## Result and discussion

### Normal condition analyzing

The result of this study showed that Intraoperative concentration of bacterial and fungal bioaerosols in indoor air of the orthopedic, internal and cesarean operating rooms was significantly higher than their preoperative concentration (p-value<0.05). Also, there was no significant difference among the bacterial and fungal concentrations in different operating rooms (p-value<0.05). Also, the significant difference was not in the bacterial concentration in different operating rooms (p-value <0.05).

### The contribution of the bacterial and fungal genera

A total of 198 bacterial and 198 fungal samples were collected for identification of airborne bacteria and fungi during the six months of the sampling period in 4 operating rooms of Shariati Hospital in Karaj. Table 1 presents the genus and a median number of bacteria observed in the operating rooms before, during and after surgery. The bacterial genera found in the operating room include S. epidermidis, S. aureus, B. subtilis, lactobacillus, diphtheria, and E. coli. Regarding the non-normality of the data distribution, Wilcoxon signed rank test was used to compare the concentration of bioaerosols before, during and after surgery.

**Table 1:** The genus and the median number of bacteria observed in the operating rooms before, during and after surgery (CFU/Plate).

Genus	Eye surgery			Orthopedic surgery			Internal Surgery			Cesarean section		
	Before	During	After	Before	During	After	Before	During	After	Before	During	After
S.epidermidis	2.20	2.60	3.40	2.46	4.00	2.61	5.00	5.50	4.75	2.00	3.50	1.50
S. aureus	0.60	0.80	1.20	0.60	0.64	0.69	0.50	0.50	0.50	ND	ND	0.50
B. subtilis	0.20	0.20	0.40	ND	0.37	0.36	0.25	0.70	0.25	0.50	ND	1.00
lactobacillus	0.40	0.20	0.40	0.40	ND	0.44	1.50	ND	0.25	2.50	1.00	0.50
diphtheriae	ND	0.20	0.50	0.64	0.64	0.64	ND	0.50	0.75	ND	4.00	1.00
E. coli	–	–	–	0.08	0.08	0.08	–	–	–	–	–	–

Table 2 presents the genus and a median number of fungi observed in the operating rooms before, during and after surgery. The concentration and number of species of fungi found were less than the observed bacteria. The fungal genera found in the operating room included Cladosporium, Aspergillus, and Penicillium. Paired *t*-test was applied to compare the concentration of bioaerosols before, during and after surgery. The results showed that the intraoperative concentration of fungal bioaerosols in the orthopedic, internal and cesarean operating rooms was significantly higher than their preoperative concentration (p-value <0.05). Also, the postoperative concentration of fungal bioaerosols in the orthopedic, internal and cesarean surgery rooms was significantly higher than their preoperative concentration (p-value <0.05). Also, there was no significant difference between the concentrations of fungi in different operating rooms (p-value <0.05).

**Table 2:** The genus and a median number of fungi observed in the operating rooms before, during and after surgery.

Genus	Eye surgery			Orthopedic surgery			Internal Surgery			Cesarean section		
	Before	During	After	Before	During	After	Before	During	After	Before	During	After
Cladosporium	1.80	2.40	1.75	0.6	2.38	2.07	1.02	1.97	2.75	0.50	1.50	2.00
Aspergillus	1.60	2.00	1.00	0.46	2.07	1.46	1.00	0.80	ND	ND	1.50	ND
Penicillium	1.00	1.00	0.40	1.54	1.00	0.76	0.50	0.80	0.50	0.50	1.00	ND

In order to better understand of bioaerosol trend, Fig. 1, Fig. 2, Fig. 3, Fig. 4, Fig. 5, Fig. 6, Fig. 7, Fig. 8 indicate the trend of bacteria count in different surgery rooms.

**Fig. 1:**
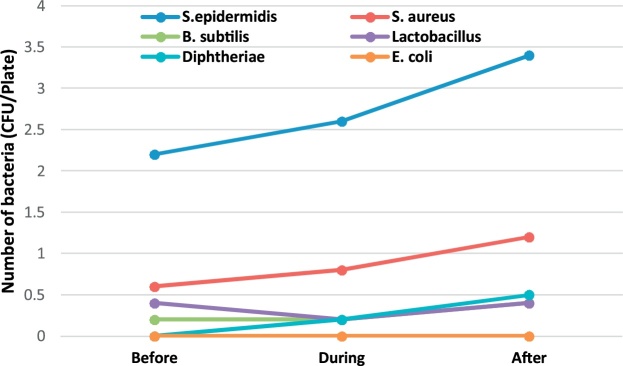
The trend of bacteria count in the Eye surgery ward.

**Fig. 2:**
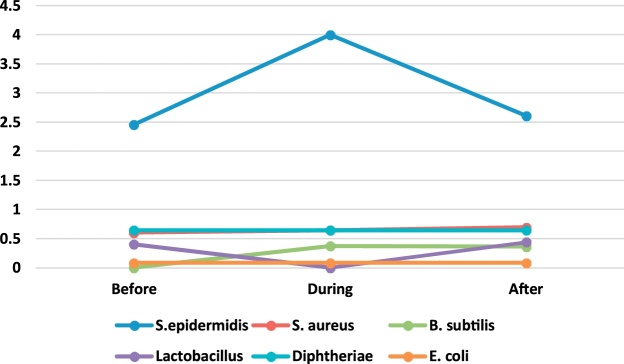
The trend of bacteria count in Orthopedic surgery ward.

**Fig. 3:**
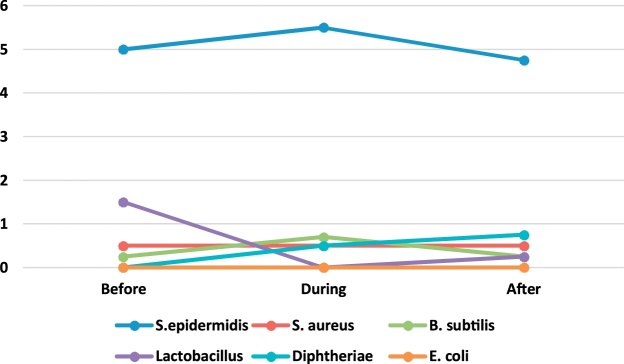
The trend of bacteria count in Internal surgery ward.

**Fig. 4:**
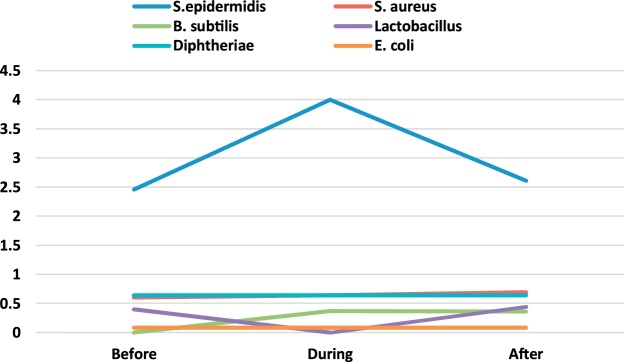
The trend of bacteria count in Cesarean section ward.

**Fig. 5:**
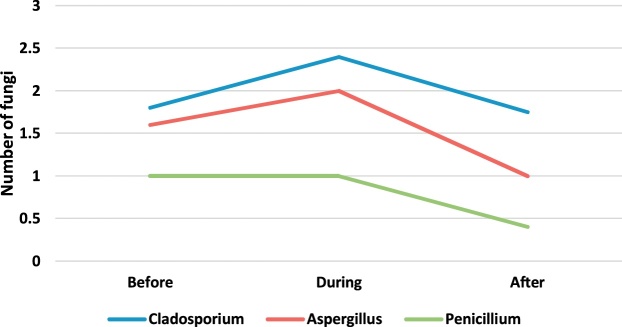
The trend of the fungal count in Eye surgery section ward.

**Fig. 6:**
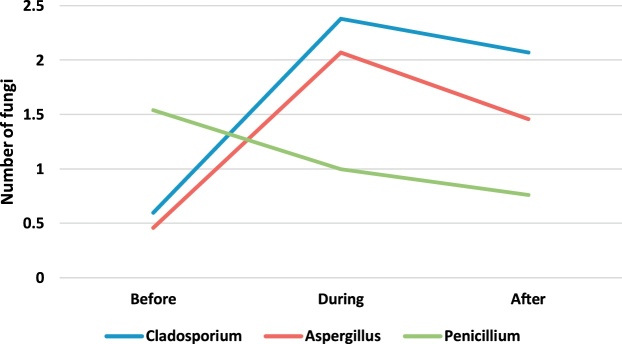
The trend of the fungal count in Orthopedic surgery ward.

**Fig. 7:**
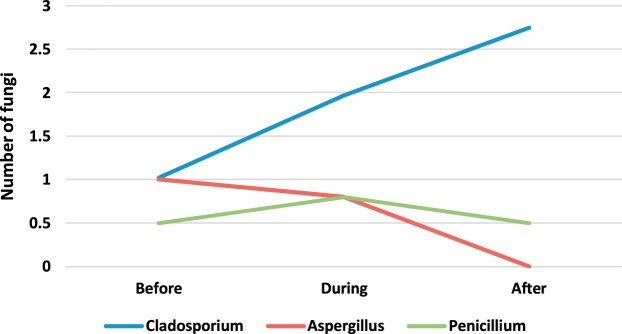
The trend of the fungal count in Internal Surgery section ward.

**Fig. 8:**
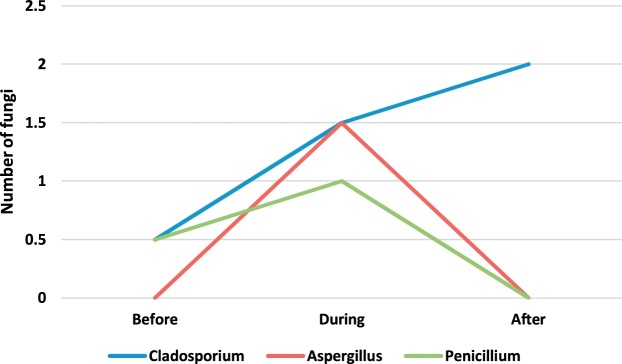
The trend of the fungal count in Cesarean section ward.

### Statistical analysis

In present work, Pearson correlation test was used to evaluate the effect of the surgical incision size on the concentration of bacterial and fungal bio-aerosol. The results of Pearson correlation test are presented in Table 3. The results showed no significant correlation between bacterial and fungal bio-aerosol concentrations and the surgical incision size (p-value> 0.1). There was also a correlation coefficient of 0.53 between the concentrations of airborne bacteria and fungus (p-value<0.05).

**Table 3:** The results of the correlation coefficient between bacterial and fungal bio-aerosol concentrations and the surgical incision size.

Parameter	Incision size		Bacterial concentration		Fungi concentration	
	correlation coefficient	p-value	correlation coefficient	p-value	Correlation coefficient	p-value
Incision size	1	0.00				
Bacterial concentration	0.10	0.64	1	0.00		
Fungi concentration	0.02	0.94	0.53	0.01	1	0.00

### Comparing the results with similar studies

The results of the present are consistent with the results of other studies, which showed that staphylococcus, diphtheria, B. subtilis, etc. made up the highest number of bioaerosols observed in hospital settings [[24], [25], [26]]. Mirzaei et al. [27] identified 17 bacterial species in the operating rooms of a hospital, which included Staphylococcus, Micrococcus, pneumococcus, E. coli, streptococcus, B. cereus and subtilis, Pseudomonas, etc. [27]. All bacterial species observed in this study (except *E. coli*) are gram-positive. The ratio of gram-positive bacteria to total bacteria observed in previous studies was reported to be more than 88% [28,29]. Gram-positive bacteria present in micro and macro environments belong to natural microbial flora of the skin, mucous membranes, and human and animal hair [30]. Also, the resistance of gram-positive bacteria is much higher than gram-negative bacteria. These bacteria can survive under adverse environmental conditions as well as closed environments [31,32]. The fungal genera found in the operating room include Cladosporium, Aspergillus, and Penicillium. The findings of this research are consistent with other studies. Several studies have reported that Penicillium and Aspergillus species are the major fungal species found in hospital settings and the main causes of hospital infections [22,28,33]. Faure et al. [34] sampled and analyzed the indoor air of 17 operating rooms for eight years. The results showed that various fungal species found in the operating room included penicillium species (28.4%), Cladosporium species (15.6%), and Aspergillus species (6.6%) [34]. One reason for it could be because these species are resistant against dehydration, which enables them to survive in hospital wards and operating rooms for a long time [20]. Also, operating room ventilation systems have been introduced as one of the sources for the release of fungal bioaerosols [35]. The results of this investigation showed that the concentration and number of species of bacteria observed in the operating rooms were more than the concentration and number of species of fungi observed. In the study on the microbial quality of the operating rooms in Turkey, Sarıca et al. [36] reported that both the number and concentration of bacterial species (10 species) was higher than the observed fungal species (7 species) [36]. Li et al. [37] studied the microbial quality of clean rooms in hospitals. They found that although no fungi were found in these class 100 clean rooms, bacteria were observed at a concentration of CFU/m^3^ [37]. This could be due to a higher number of bacterial bioaerosols in natural resources such as the soil and air of highly vegetated areas [38,39]. On the other hand, more fungal bioaerosols can be deposited due to their larger diameters, which can change the ratio of bacterial and fungal species deposited [31]. Sautour et al. sampled the number of bioaerosols using two active and passive methods and found a significant difference in terms of number and types of bioaerosols collected by these two methods [40]. This difference can be due to the various mechanisms used in the two methods for the trapping of bio-aerosols in such way that only those bio-aerosols, which have gravitational sedimentation rate sufficient to be deposited in the sampling plates, are trapped in the passive sampling method [19,41]. Therefore, it is impossible to compare the results of studies using different sampling methods. However, the passive sampling method seems to be appropriate to determine the relative concentration of bioaerosols in hospital settings [42]. Sudharsanam et al. [41] presented a set of suggested guidelines for passive sampling of bioaerosols in hospital settings. According to these guidelines, hospital wards with < 20 CFUs and > 50 CFUs should be categorized as low and high contaminated wards respectively. Also, the bacterial and fungal bioaerosols concentration in the surgical wards should be < 1 CFUs and >10 CFU, respectively [42]. When the results of this research are compared with these guidelines, it becomes clear that the concentration of bacterial bioaerosols in the operating rooms is within acceptable limits, but the concentration of fungal bioaerosols in the operating rooms is not within acceptable limits. In a study, Rocha et al. [42] compared the results with the criteria of the Spanish Union Hospital Engineering and the Canadian Department of Health and Welfare. They found that although most operating rooms are "clean" for bacterial bioaerosols, several operating rooms still exceed the "non-contaminated" range. Majority of operating rooms were "very clean" and "clean" in terms of fungal bioaerosols [43]. The results also showed a weak and insignificant correlation between the surgical incision size and the concentration of bacterial and fungal bioaerosols. This means that the surgical incision size, whether small or big, does not affect the concentration of bioaerosols in the operating rooms. However, according to the results of the present study, suggesting the intraoperative concentration of bioaerosols, the hypothesis suggesting the lack of effect of surgery on the concentration of bioaerosols is thus rejected. So, it can be deduced that, by combining these two results, surgery increases the bioaerosols concentration, but the small and big incision size does not affect the concentration of bioaerosols [43,44]. On the other hand, the high concentrations of bacteria and fungi deposited in the indoor air of the operating rooms indicate a high transmission and incidence potential for hospital infections in these places [44]. The microbial species found in this study can cause serious infections and complications. The results of the present study seem to show that in addition to the sterility of the operating room equipment, the indoor air of these places should also be considered as one of the major causes of the occurrence of hospital infections [45,46].

## Conclusions

Concentrations and types of bacterial and fungal bioaerosols were determined in four operating rooms of Shariati Hospital in Karaj. Also, the present evaluated the relationship between bioaerosols concentration and surgical incision size. The intraoperative concentration of bacterial and fungal bioaerosols in the orthopedic, internal and cesarean operating rooms was significantly higher than their preoperative concentration. Also, there was no significant difference between bacterial and fungal concentrations in different operating rooms. On the other hand, the results showed no significant correlation between the concentration of bacterial and fungal bioaerosols with the surgical incision size. The results of the present investigation can be useful in determining the dominant microbial species in the operating rooms and their associated infection risk. Carrying out surgical procedures on bioaerosol-containing settings can lead to the incidence of hospital infection. The results indicated that the gram-positive bacteria and the Penicillium and Aspergillus fungi are the most frequent species. These species have more resistance in adverse conditions, and more consideration should be given to the disinfection of the operating rooms.
